# Nanocellulose Bio-Based Composites for Food Packaging

**DOI:** 10.3390/nano10102041

**Published:** 2020-10-16

**Authors:** Francisco A. G. S. Silva, Fernando Dourado, Miguel Gama, Fátima Poças

**Affiliations:** 1 Centre of Biological Engineering, University of Minho, Campus de Gualtar, 4710-057 Braga, Portugal; francisco.agss@gmail.com (F.A.G.S.S.); fdourado@deb.uminho.pt (F.D.); 2 Escola Superior de Biotecnologia, Laboratório Associado, CBQF–Centro de Biotecnologia e Química Fina, Universidade Católica Portuguesa, Rua Diogo Botelho 1327, 4169-005 Porto, Portugal; mpocas@porto.ucp.pt

**Keywords:** nanocellulose, bio-based, composite, food requirements, packaging, barrier properties

## Abstract

The food industry is increasingly demanding advanced and eco-friendly sustainable packaging materials with improved physical, mechanical and barrier properties. The currently used materials are synthetic and non-degradable, therefore raising environmental concerns. Consequently, research efforts have been made in recent years towards the development of bio-based sustainable packaging materials. In this review, the potential of nanocelluloses as nanofillers or as coatings for the development of bio-based nanocomposites is discussed, namely: (i) the physico-chemical interaction of nanocellulose with the adjacent polymeric phase, (ii) the effect of nanocellulose modification/functionalization on the final properties of the composites, (iii) the production methods for such composites, and (iv) the effect of nanocellulose on the overall migration, toxicity, and the potential risk to human health. Lastly, the technology readiness level of nanocellulose and nanocellulose based composites for the market of food packaging is discussed.

## 1. Introduction

Packaging plays an essential role in the food supply chain, protecting and containing food from the processing and manufacturing stages, along with distribution, handling and storage, until it reaches the final consumer. Currently, food packaging accounts for the largest share in the total packaging sector (85%). The global packaging market revenues increased from $42.5 billion in 2014 to nearly $48.3 billion by 2020 [1]. Plastics are the most used packaging materials, bearing lightweight, with good processability, low production cost and outstanding mechanical and barrier properties [2]. The plastic packaging market has been expanding with a growth rate of 20–25% per year [1]. However, there is an increasing concern regarding the massive use of petroleum-based plastics, which are used for a short period of time but then take centuries to degrade. Most synthetic plastics used in packaging are recyclable. In the scope of the European Strategy for Plastics in a Circular Economy, the Directive (EU) 2019/904 was published to prevent and reduce the impact of single use plastic products on the environment, in particular the aquatic environment, and on human health, as well as to promote the transition to a circular economy [3]. Additionally, the Directive (EU) 2018/852 sets a minimum of 70% by weight of all packaging waste as target for recycling and a minimum of 55% for plastic, by the end of 2030 [4]. However, in some countries, the recycling rates are still rather low: in 2017, the European Union had a plastics packaging recycling rate of 41.7% [5]. Furthermore, mechanical recycling, the most common process used, impacts the final properties of the plastics [6]. Since 1950, about 6300 million tons of plastic waste have been generated, of which 4977 million accumulated in landfills and waterways [7]. Another emerging problem are the so called microplastics (< 5.0 mm) that are present in the air, water and soil [6], with harmful effects to both terrestrial and marine ecosystems.

Therefore, industry and academia have been working on the development of renewable and sustainable alternatives with competitive features [8]. Biopolymers, while biodegradable and highly available, often have inferior performance than their petroleum-based counterparts. To improve their performance, composite technology has emerged as an approach to blending biopolymers with different properties, leveraging on the best properties of each individual component. In the global biodegradable polymer market, the revenues are expected to grow from $3.1 billion in 2016 to $7.1 billion by 2021, an annual growth rate of 18% [1].

Vegetable cellulose and its derivatives are widely used for paper production, pharmaceutical compounds, textiles and packaging [9]. Paper, paperboard and Cellophane^TM^ are examples of cellulose-based materials used traditionally in food packaging. More recently, plant nanocellulose (NC) (which includes nanocrystals (CNCs) and nanofibrilated cellulose (NFCs)) became industrially available, offering unique characteristics such as high specific surface area, and consequently high concentration of active groups for surface modification, and the ability to improve the mechanical performance of the nanocomposites [9,10,11,12,13,14]. Nanocellulose has been widely studied for the application in textile [2], optical electronic devices [15], food industry [9], biomedicine [8,16] and to a lower extent in papermaking [17]. Lately, research and development communities have sought the development of nanocellulose-based materials for food packaging applications [18,19], focusing mainly on improving their mechanical and barrier properties, by using different nanocelluloses, production processes and matrix biopolymers [20]. Nanocellulose is also a trending material as a support in active and intelligent packaging for additional functionalities such antimicrobial and antioxidant properties, as well as a component in sensoring systems [21,22,23]. However, like any other food contact material, nanocellulose in food packaging applications raises potential safety concerns.

## 2. Food Packaging Requirements

Packaging has become essential in modern life and its use has increased over time. The main goal of packaging is to contain food in a cost-effective way, while satisfying industry and regulatory requirements and consumer expectations, keeping food products protected from the external environment [2]. For the majority of food products, the protection afforded by the package is an essential part of the preservation process. The requirements of a packaging system intended for fresh, frozen, dehydrated, thermal or aseptic processed products, depend on the: (i) intrinsic properties of the food product, such as water activity and oxidation potential that determine their perishability; (ii) extrinsic factors, namely storage temperature, relative humidity and exposure to light, and finally (iii) the required shelf-life. All these factors must be taken in account when specifying the required barrier ability to water vapour, oxygen and other gases, including aromas and light. The physical and mechanical properties are also important [24] during processing, packaging operations and handling through the supply chain. Packaging geometry (surface area to volume) and the material thickness are variables that largely affect both the barrier and the physical performance of the package. Table 1 presents the requirements regarding moisture and oxygen barrier for several foods (information adapted from [25]) for materials commonly used for this application.

**Figure 1:**
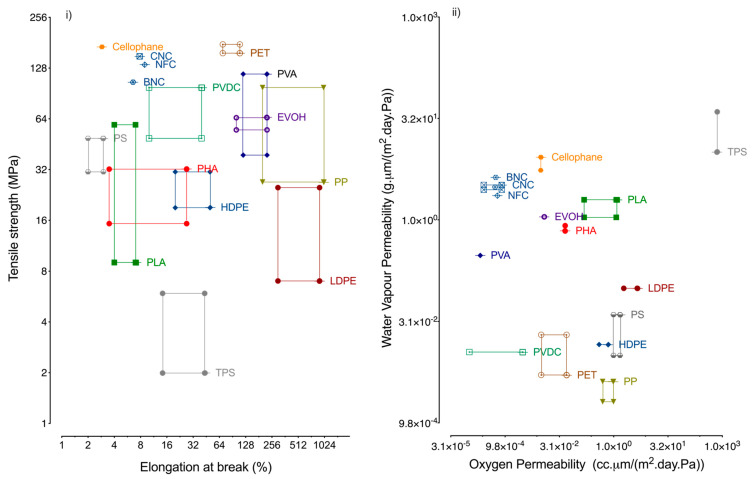
Properties of synthetic and bio-based polymers: (**i**) Tensile strength (MPa) vs elongation at break (%); (**ii**) Water vapour permeability vs Oxygen permeability; the values, obtained at 23–25 °C and normalized in terms of relative humidity (50% RH), were calculated from [2,19,29,30,31,32,33,34,35,36,37,38,39,40,41,42,43]. PET-Polyethylene Terephthalate; PP-Polypropylene; LDPE-Low density polyethylene; HDPE- High density polyethylene; PS-Polystyrene; PVDC-Polyvinylidene chloride; PVA-Poly vinyl alcohol; EVOH-Ethylene vinyl alcohol; PLA-Poly lactic acid; PHA-Polyhydroxyalkanoates TPS-Thermoplastic starch.

It is recognised that petroleum-based plastics have great thermomechanical and barrier properties, as well as light weight and low production cost [26], yielding a hard to beat overall performance (Figure 1); they are commonly used in food packaging as indicated in Table 1.

The environmental impact of non-biodegradable plastic materials and the increasing need for a more sustainable use of packaging and plastics in particular, are ever-growing global concerns. Solutions to reduce and in some cases to replace those materials are on top of research efforts. Biopolymers such poly lactic acid (PLA), polyhydroxyalkanoates (PHA) and thermoplastic starch (TPS) have been sought as alternative solutions [17,27]. The most widely exploited one is PLA [18], which is mainly used in packaging applications. It is used as films or thermoformed or injected packages for relative short-term and mild temperature contact conditions, such as fresh salads and beverage drinks, because of its low resistance to temperature. One major limitation commonly referred is the high price and commercial shortage, as compared to conventional plastics.

Corn is presently the major raw material used in the production of PLA but a second-generation feedstock is under development. Although generally considered a biodegradable material, PLA is actually poorly degradable under simulated ocean and soil conditions, only being compostable at high temperatures [28]. PHAs, and in particular poly-(3-hydroxybutyrate) (PHB) is one of most widely studied biopolymers, the easiest to produce, and is regarded as an alternative to polypropylene (PP) for food packaging [29], although with much less commercial applications. Starch, is the second most abundant biopolymer in nature [30]. Thermoplastic starch (TPS) in particular has potential for food packaging application, in spite of its poor mechanical and barrier properties (Figure 1). An important commercial application is that of bags for non-packaged fruits and vegetables and as shopping bags, in blends with fossil-based polymers. However, the bio-based materials available lack the performance to fulfil the most demanding specific requirements (Table 1) for food packaging [25,31] and apart a few exceptions, commercial applications are yet not volume comparable. In Figure 1, the mechanical (tensile strength, elongation at break) and barrier properties (water vapour permeability and oxygen permeability of conventional plastics and biopolymers are compared. In general, biopolymers have lower elongation at break values and higher water vapour permeability than conventional plastics (Figure 1).

NC has received an exponential interest as a component for food packaging purposes and its properties include a high stiffness (comparable to polyethylene terephthalate (PET)) and a low oxygen permeability (comparable to ethylene vinyl alcohol (EVOH)), as depicted in Figure 1. Despite the good mechanical and oxygen transfer properties in dry conditions, the moisture barrier ability of nanocellulose is one of the poorest when compared to petroleum-based plastics and biopolymers (Figure 1).

In order to improve the performance of the mentioned biopolymers, up to a level compatible with food packaging applications, nanocomposite technology has been regarded as an option, consisting on the combination of two or more materials, to enhance the overall properties of the composite. However, the outcome of NC mixtures with other polymers is not straightforward, as it depends on the ability of NC to interact with the other materials, in particular through hydrogen bonding.

## 3. Nanocellulose Applications in Food Packaging

Nanocellulose can be isolated from plant sources or produced through microbial fermentation [44]. The extraction/production method influences the nanocellulose characteristics, namely crystallinity, degree of polymerization (DP), fibre diameter and length, which are key in determining the mechanical and barrier properties [13]. Regardless of the cellulosic source, the chemical formula of the biopolymer is (C_6_H_10_O_5_)_DP_.

Due to its abundance, renewability and degradability, physical-chemical and morphological properties NC has shown outstanding potential to reinforce bio-based materials [9,10,11]. Plant cellulose has already a long history of use in food packaging, namely paper and board, Cellophane^TM^, and modified cellulose derivatives such as cellulose acetate, methylcellulose (MC), hydroxypropyl cellulose (HPC), hydroxypropyl methylcellulose (HPMC) and carboxymethylcellulose (CMC) [45].

### 3.1. Nanocellulose Sources

The nanocellulose, nanofiber, or nano-structured cellulose is characterised by the nanosize of the fibres (<100 nm) in at least one dimension. NC features high crystallinity, high degree of polymerization, high mechanical strength, low density, biocompatibility, non-toxicity and biodegradability [44,46]. The high amount of hydroxyl groups enables its chemical surface modification. The NC production may be divided into bottom-up and top-down methods. Examples of top-down methods are steam explosion, enzyme-assisted and acid hydrolysis (using sulfuric and hydrochloric acids), followed by mechanical treatments (high pressure homogenization, microfluidization and cryocrushing) [44,47]. The NCs obtained by these methods are the: (i) nanofibrillated cellulose (NFC), with a diameter between 5 and 20 nm and a length between 2 and 10 micrometers and (ii) cellulose nanocrystals (CNC), which refer to the most crystalline structures obtained by hydrolysis [44,47,48]. There are significant differences between NFC and CNC. NFC are longer and flexible filaments, alternating crystalline and amorphous regions, while CNC has a rod-like shape and are more crystalline. Crystalline domains in NFC have similar dimensions of CNC. Examples of bottom-up methods are the production of bacterial nanocellulose (BNC) and cellulose from tunicates.

Vegetable NC has been obtained from a wide variety of sources, namely, pine, coconut husk fiber, mengkuang leaves (*Pandanus tectorius*), raw cotton linter, barley wastes, tomato peels, garlic straw residues, forest residues, corncob residue, industrial waste cotton, cassava root bagasse and peels, sugar palm fibers, corn straw and agro-industrial residues [48]. The plant source (fibre dimensions, structure of the cell wall, relative percentage of cellulose, hemicellulose and lignin) and the extraction method will influence the final NC purity and properties.

Biotechnological nanocellulose, BNC, consist of pure cellulose nanofibers extruded by certain acetic acid bacteria (e.g., *Komagataeibacter xylinus*), forming a 3D nanofibrillar matrix during static fermentation. Although chemically identical to vegetable NC, BNC has higher DP and longer fibres [49]. Its characteristics are influenced by the type of strain and the fermentation conditions [45].

### 3.2. Nanocellulose Based Composites

Nanocellulose (NC) has been used both as a coating and a filler, to produce nanocomposites for food packaging. Understanding the contribution of each component and their interaction is necessary to optimise the overall performance of the composite.

Coating can be defined as the surface application of a thin film onto a substrate, producing a multilayered material [14]. Numerous food packaging applications require good barriers for oxygen and grease, such as fast food, pet food and bakery products [50]. The use of NC based coatings on paper and paperboard has been reported [50,51,52,53,54]. In general, the coating of paperboard with NC based layers reduced the oxygen permeability and improved the grease resistance, but a high water vapour permeability was still observed [50,51,52,53,54]. To reduce the high water vapour permeability of NC, combination with, for example, polypyrrole and PLA were reported [53,55]. Polypyrrole particles were added to NFC suspension before coating the paperboard. A decrease in oxygen permeability and an increase in the mechanical properties were obtained after coating [53]. In another approach, a multilayer coating onto paperboard was produced using PLA as the outer layer, while the CNC was the intermediate one, together yielding low oxygen permeability and water vapour permeability [55]. Transparent multilayer films were produced from NFC thin layers coated on amorphous PLA and semi crystalline PLA substrates. A strong adhesion between the layers was obtained, which led to an improved mechanical performance [56]. Another interesting example is the coating of NFC onto multilayer bio high-density polyethylene (HDPE) film. The NFC layer on the bio-HDPE led to a decrease in oxygen permeability (both at 0% RH and 80% RH) and improved grease resistance. The NFC layer loading did not compromise the already good water vapour barrier of neat bio-HDPE [57].

Another strategy is to use nanocellulose as a filler of a polymeric continuous phase. Biopolymers such PLA, PHB and TPS have been explored for that purpose. However, the compatibility between matrixes of different hydrophobicity must be enhanced and this is many times performed through NC modification. PLA based composites reinforced with low concentrations (0.5–1.0%) of BNC fibres had higher mechanical properties (higher tensile strength and Young modulus) and lower water vapour permeability, in comparison to NFC-PLA composites and neat PLA films [58]. The Young modulus, crystallinity and thermal stability improved with BNC loading [59]. However, the water vapour permeability increased with higher NFC loadings [60]. It should be highlighted that the performance depends largely on the dispersion of the nanofibres within the matrix of PLA. CNC could be efficiently dispersed in PHB and PLA blends, improving interfacial adhesion between PLA and PHB, leading to improved mechanical properties, thermal stability and crystallinity of the composite [61,62]. The combination of PHB and CNCs reduced the PLA oxygen permeability ca 24% down to 23.3 cm^3^mm m^−2^day^−1^ even with the addition of a plasticizer to the system, highlighting the positive interaction between all the blend components [62]. The blend of nanocellulose (either NFC, CNC or BNC) with TPS improved the mechanical performance over neat TPS film. However, these NC-TPS composites still showed poor moisture barrier properties [63,64,65,66,67,68,69].

**Figure 2:**
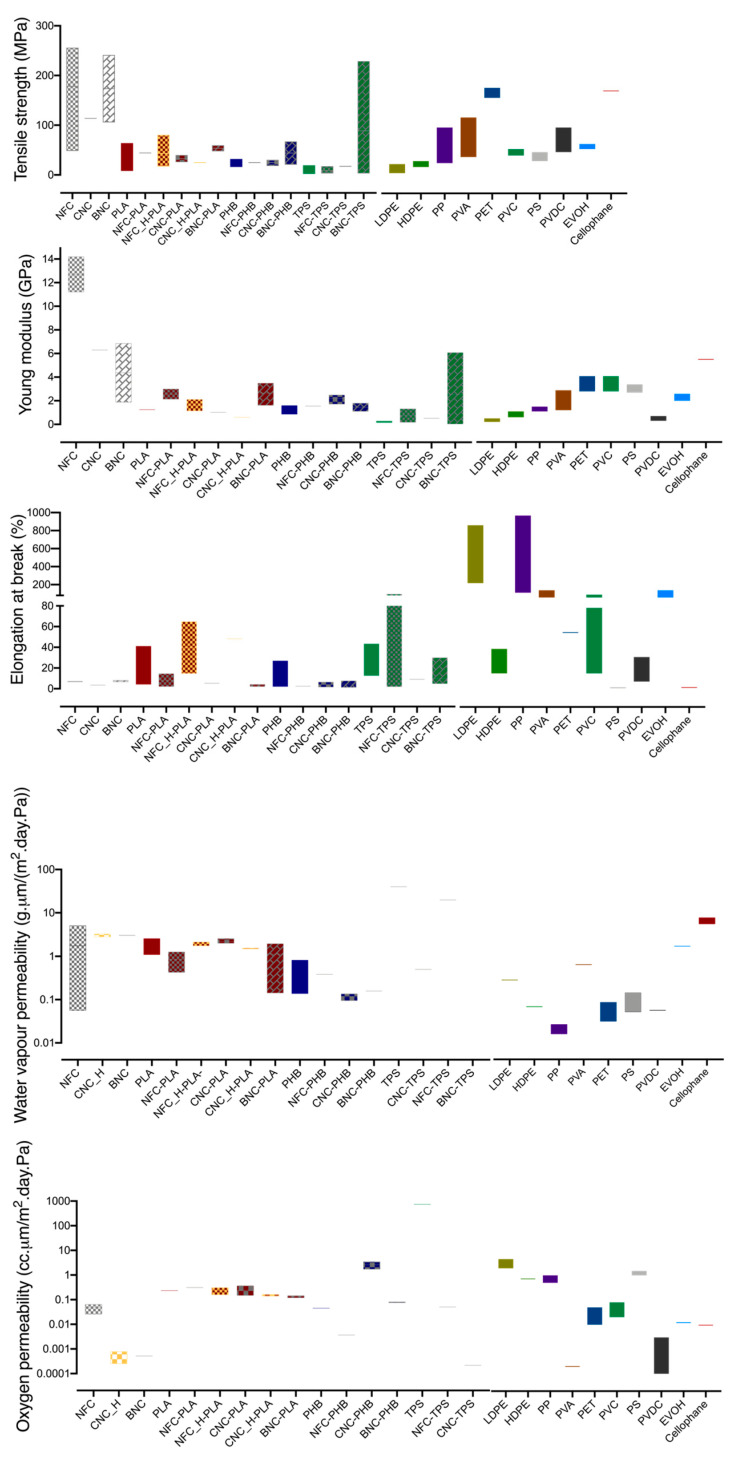
Mechanical and barrier properties of nanocellulosic based composites and petroleum-based plastics [34,35,37,41,58,59,60,62,65,66,67,68,69,70,71,72,73,74,75,76,77,78,79,80,81]. PET—Polyethylene Terephthalate; PP-Polypropylene; LDPE—Low density polyethylene; HDPE-High density polyethylene; PS-Polystyrene; PVDC-Polyvinylidene chloride; PVA-Poly vinyl alcohol; EVOH-Ethylene vinyl alcohol; PLA-Poly lactic acid; PHA-Polyhydroxyalkanoates TPS-Thermoplastic starch; NFC-nanofibrilated cellulose; CNC-cellulose nanocrystals; BNC-bacterial nanocellulose; NFC_H-hydrophobized nanobribrilated cellulose; CNC_H-hydrophobized cellulose nanocrystals; Modified NCs were colored orange.

Figure 2 shows the values of the mechanical and barrier properties of nanocellulosic composites found in the literature. Each column (that refers to a particular material or combination of materials) represents the value range for a specific property (Y axis), regardless of the production method, biopolymer concentration used and NCs modification. The main goal is to provide an overall comparative view of the mechanical and barrier performance of NC based composites with conventional plastics. NC increases the stiffness of all composites (either with PLA, PHB and TPS), since it led to an increase in Young modulus and a substantial decrease on the elongation at break (Figure 2).

The water vapour permeability of PHB, TPS and PLA tends to decrease with the NC incorporation (either NFC, CNC or BNC). This is observed for low nanocellulose concentrations (below 5%), which can be well distributed in the relatively hydrophobic matrix, hindering the transmission of water vapour through the composite. However, agglomeration may occur for higher NCcontent, leading to null or even reverse effect on water vapour permeability. Concerning the oxygen permeability, there is no significant effect of NC on PLA based composites. NFC and BNC decrease the oxygen permeability of PHB and TPS based composites, while CNC substantially reduces it (Figure 2). Despite the overal reduction observed, it is important to consider the relative humidity conditions used in the oxygen permeability measurements. Often these are performed in dry conditions and RH values higher than 50% have a strong interaction with the nanofibres network leading to a substantial increase of oxygen permeability (Figure 2).

In short, the incorporation of nanocelluloses (BNC, CNC and NFC) improves the mechanical properties (increasing stiffness) and decreases the water vapour permeability of PLA, PHB and TPS, due to the enhanced structural properties of the final material. This beneficial interaction brings these composites closer to the requirements in food packaging. However, these improvements are still insufficient, since high oxygen and water vapour permeabilities are still observed (higher than that of the petroleum-based plastics) (Figure 2). Hence, despite the significant advancements, these composites still need improvements, especially regarding water vapour barrier, since food products usually have high water activity and the surrounding environment also has a relative humidity of around 50%.

### 3.3. Modification of Properties, Processability and Functionalization of Nanocellulose

Nanocellulose fibres can be modified to: (i) enhance their interaction with the matrix phase in composite processing, (ii) improve the intrinsic properties of the fibres and (iii) provide attractive functions for specific applications, for example active and intelligent packaging, to ultimately benefit the quality and safety of the food product. In this latter case, surface functionalisation can be performed to provide antimicrobial and antioxidant properties, to develop sensor systems and to provide sites for specific interaction with different chemical agents. Several approaches as silane grafting, acetylation, alkylation, esterification, 2,2,6,6-tetramethylpiperidin-1-oxyl (TEMPO) oxidation, carboxymethylation and the use of nanoparticles were used to modify nanocellulose with the abovementioned objectives [82,83,84,85,86,87,88].

#### 3.3.1. Modifications to Improve the Compatibility between Nanocellulose and Plastic Matrixes

Several NC modification strategies aiming to overcome the lack of compatibility between NC and hydrophobic polymers and improve the dispersion of the fibers have been reported. CNC grafted with *n*-octadecyl isocyanate, phenylacetic acid or benzylacetic were successfully dispersed in a PLA matrix [34,72]. Also, the use of a cationic surfactant, such as lauric arginate, allowed to tailor the surface polarity of CNC, improving its dispersion in the PLA matrix [89]. Other approach is the incorporation of non-ionic surfactants such as nonylphenol-based phosphate esters [60] or the plasticiser acetyl tributyl citrate [63] for the improvement of the interfacial adhesion between CNC and PLA-PHB matrix phase. Well dispersed CNC increased the PLA crystallinity, improved the processability and thermal stability of the nanocomposite [62,63]. The tensile strength of the CNC-PLA composite was improved by 10 MPa with the modification with isocyanate and the water vapour permeability was lower than the one of ungrafted CNC composite [34]. All the reported approaches allowed an improvement of the mechanical performance and moisture barrier, in comparison to unmodified CNC-PLA composite [34,72,89].

#### 3.3.2. Modifications to Improve Moisture Resistance

Due to its hydrophilic nature, the oxygen barrier effect of cellulose-based materials is greatly reduced at high moisture, limiting the application of these materials in the food sector. For example, carboxymethylated nanofibrilated cellulose films presents low oxygen permeability (0.009 (cm^3^·µm)/(m^2^·day·kPa)) at 0% RH, within the same range of EVOH (0.01–0.1 (cm^3^·µm)/(m^2^·day·kPa)). However, at 80% RH the oxygen permeability of the same films increases exponentially to 30.6 (cm^3^·µm)/(m^2^·day·kPa) [90]. The permeability of EVOH also increases with the RH but at a lower extent and depending on the relative percentage of ethylene in the copolymer. Thus, the control of moisture resistance of NC is a means to control the oxygen permeability.

One of the most studied aspects is the hydrophobization of cellulose to improve the moisture resistance, while improving the compatibility with the (hydrophobic) polymer matrices [73,82,83,91,92,93,94]. However, making NC more hydrophobic, also render it less able to block oxygen. Hence, a fine-tuning balance is needed to fit each application. The treatment of BNC and vegetable microfibrilated cellulose with several anhydrides (acetic, butyric, hexanoic and alkenyl succinic anhydrides) and hexanoyl chloride suspended in an ionic liquid (tetradecyltrihexylphosphonium bis(trifluoromethylsulfonyl)imide) led to their surface hydrophobization [82].

The surface modification of BNC via esterification with organic acids (acetic, hexanoic and dodenoic) was reported. The longer the length of the hydrocarbon chain with which the BNC was modified, the more hydrophobic was the BNC surface [83].

A two-step modification procedure, based on periodate oxidation followed by reductive amination, allowed the production of CNCs from butylamino-functionalized pulps [41]. Tert-butylamino functionalized CNCs showed enhanced mechanical properties, with an increase of 18% in tensile strength and 10% in Young modulus. The functionalized CNC films showed an oxygen permeability increase from 0.25 at 50% RH to only 5.9 (cm^3^·µm)/(m^2^·day·kPa) at 80% RH.

NFC was surface modified by the adsorption of a cetyltrimethylammonium bromide (anionic surfactant) and cetyltrimethyl-, didodecyl- and dihexadecyl ammonium bromide (cationic surfactants), leading to more hydrophobic surfaces [95,96].

#### 3.3.3. Modifications to Provide Active and Intelligent Functionality

Active and intelligent packaging are concepts that became very attractive for researchers and a very high number of related works are published every year. Active packaging is a system that absorbs or releases substances in order to extend the food shelf life, providing for example antimicrobial and antioxidant properties. Intelligent packaging monitors the condition of packaged food or the surrounding environment providing information, for example on the freshness of the food. Nanocellulose is inert with respect to these functions, but is an excellent support for substances that may play an active or intelligent role in the food packaging system. Combinations of nanocellulose with different active agents have been reported, as for examples: flavonoid silymarin (SMN) [86], ferulic acid and derivatives [97], tannins [98], titanium dioxide (TiO_2_) [99], silver [100], lactoferrin [101], and sorbic acid [102].

BNC composite films with spherical flavonoid silymarin (SMN)-zein nanoparticles, prepared by impregnation, showed effective antioxidant properties, which were maintained for at least 72 h, due to the slow release of the active component. The antimicrobial activity of the films showed an inhibition ratio of 60, 20 and 30% for *Staphylococcus aureus*, *Escherichia coli* and *Pseudomonas aeruginosa*, respectively. The system was further tested with salmon fish, showing quality indicators of thiobarbituric acid reactive substance assay values 40% lower and total volatile basic nitrogen values 30% lower than those of the control [86].

Arabinoxylans-based nanocomposite films containing 50% nanofibrillated cellulose, prepared by solvent casting, were functionalized with ferulic acid and feruloylated arabinoxylo-oligosaccharides, showing antioxidant activity up to 90% in the 2,2-diphenyl-1-picrylhydrazyl hydrate assay. The films also showed bactericidal effects, with 3–log CFU mL^−1^ reduction against *Staphylococcus aureus*, bacteriostatic activity against *Escherichia coli* and antifungal activity towards the polymorphic fungus *Candida albicans* with 1.1–log CFU mL^−1^ reduction [97].

Vilela (2019) prepared BNC films with antimicrobial activity and with potential for application as sensors that monitor food humidity levels. A bactericidal activity against *Staphylococcus aureus* (4.3–log CFU mL^−1^ reduction) and *Escherichia coli* (1.1–log CFU mL^−1^ reduction) was reported. The films were prepared by in situ free radical polymerization of sulfobetaine methacrylate within the wet BNC nanofibrous network and in the presence of poly(ethyleneglycol) diacrylate as cross-linking agent, yielding poly (sulfobetaine methacrylate), a widely known zwitterionic polymer consisting of a trimethylammonium cation and a sulfonate anion [23].

Tannins-NFC films were produced by an *in-situ* technique, the active tannin being added to the initial dispersion of cellulose following mechanical fibrillation of the mixture. The film showed antioxidant capacity persisting after 2 days of contact in water. This functionality can be applied on the design of films with controlled release of the active component in order to suit the application of the films for foods with high water activity [98].

Composites of CNC-wheat gluten incorporating TiO_2_ nanoparticles (CNC 7.5%/0.6% TiO_2_) were prepared and applied as coatings on commercial unbleached paper. The coating was applied in 1 to 3 layers, intercalating with drying steps. The antimicrobial activity was assessed using the viable cell counting method after UV-A light exposure. Results indicated that the reduction of viable bacteria depended on the number of coating layers and on the time of exposure to UV-A light. The reduction varied from 40% to complete inactivation for *S. aureus* and *E. coli*, with exposure times from 1/2 to 2 h. Complete inactivation of yeast (*Saccharomyces cervisiae*) was achieved with the minimum conditions [99]. The inactivation effect of TiO_2_ nanoparticles was explained by the capacity to induce reactive oxygen species (ROS) under UV-A radiation.

The incorporation of silver nanoparticles (AgNPs) into BNC was performed by mixing a AgNO_3_ solution with a wet ground BNC slurry, followed by chemical or UV light reduction. The AgNP/BNC slurries were then mixed with PVA solutions to form active composite films by solvent casting. The antimicrobial activity against *E. coli* was observed for both films produced by the two methods, with up to 7 and 3 log CFU/mL reductions in liquid medium and on raw beef, respectively. The antimicrobial effect was also tested in inoculated beef showing high antimicrobial ability. The film obtained by UV reduction presented a relatively higher antimicrobial activity than the one from chemical reduction [100].

Lactoferrin-BNC films were prepared by immersing never-dried BNC films in phosphate buffer saline with lactoferrin [101], followed by drying, and assessed as edible antimicrobial packaging. These films, were previously highly contaminated with either *E. coli* or *S. aureus,* and immediately used as wrappings of fresh sausages as a model of meat products, to evaluate their efficiency under direct contact with a perishable food. The modified films showed a higher reduction (93.6% reduction) of viable *E. coli*. For *S. aureus*, inhibition reached 39.7% with the modified films, whereas *S. aureus* grew in the control group (BNC).

Sorbic acid is a food additive (E200) generally used to inhibit the growth of moulds (also mycotoxin-forming moulds), yeast and some bacteria. The additive was incorporated in BNC composite films by direct addition to a PVA and powdered BNC dispersion, following by casting. Monolayer and three-layer films were produced, where the sorbic acid containing PVA/BNC film was coated in both faces with BNC neat membranes, used to control the release rate. The antimicrobial effect was tested against *E coli* with results mainly influenced by the amount of sorbic acid released and by the water solubility of the films [102].

The functionalization of nanocellulose for intelligent food packaging has also been reported, in particular for developing freshness indicators, with the goal of food spoilage detection. Freshness indicators typically measure changes in pH or gas composition inside the packaging and these changes are translated into a colour response, which can be easily measured and correlated with the freshness of the food [21]. Kuswandi et al. [103] and Peng Lu et al. [104] developed freshness indicators with BNC-methyl red and TEMPO mediated NFC hydrogel with a mixture of bromothymol blue/methyl red, as indicators. The composites reacted to the amount of volatile biogenic amines and CO_2_ levels were found to increase with the spoilage of chicken, showing a colour change as a consequence of the pH modification.

### 3.4. Nanocellulose Based Composites Processing

Regardless of the type of NC, the conditions used to process the components and the composite production method are essential to achieve the dispersion of the fibers in the main matrix, which is critical for the performance of the final material. Several methods of processing nanocellulosic composites are mentioned in the literature [105], including:

- Solvent casting
- Melt processing
- Impregnation
- Layer-by-layer assembly
- Coating
- All-cellulose composites

#### 3.4.1. Solvent Casting

The most well-known and simple method is solvent casting, where the nanofibers are added to the polymer suspension. The suspension is stirred at a defined temperature in a reactor, in order to get a homogenous nanoscale suspension before casting onto a suitable surface with controlled thickness [81]. Evaporation at room temperature, vacuum oven drying, hot pressing and compression moulding are examples of different draining and drying procedures referred in literature, which can affect the final properties of the composite [105,106,107].

Most of the studies reported with solvent casting involve some degree of modification of the nanocellulose (surface functionalization, surfactants or emulsion systems, as described in Section 3.3) for a better dispersion in the hydrophobic matrix [106]. The solvent used is determined by the hydrophobicity/hydrophilicity of the matrix phase. For PLA, solvents such as chloroform and acetone are used [34,60]. For TPS and PVA matrices, the solvent is water [36,65,107].

**Figure 3:**
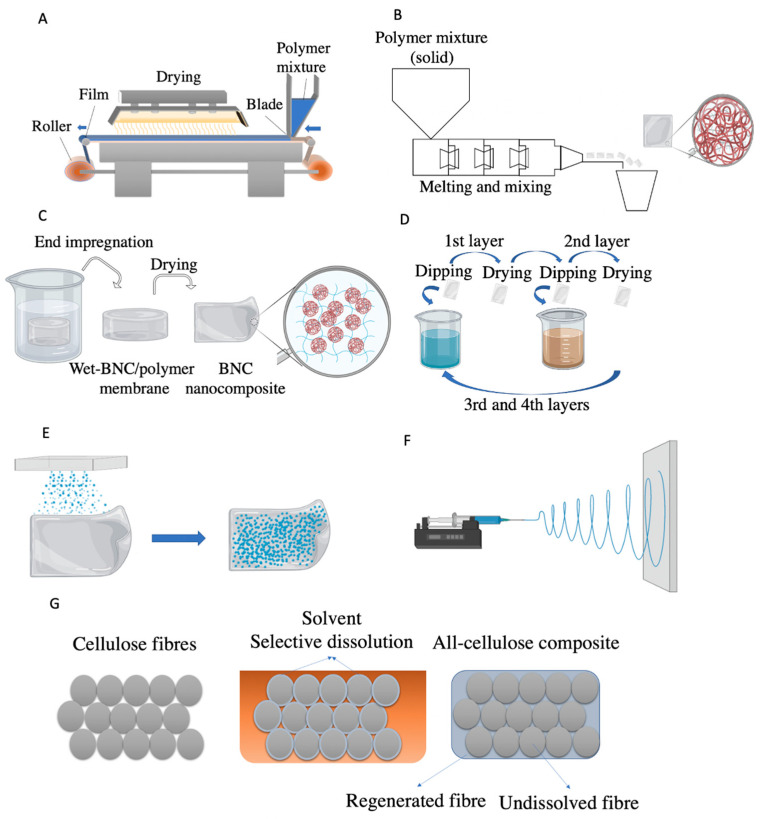
(**A**) Solvent casting; (**B**) Melt mixing; (**C**) Ex-Situ BNC Impregnation; (**D**) Layer by layer; (**E**) Coating; (**F**) Electrospinning; (**G**) All-cellulose composites; (**C**–**F**) created in bioRender.com; (**A**), (**B**) and (**G**) created in Power point.

The thickness and roughness control are crucial to maintain the quality of the film obtained by casting. Pilot scale plants currently use blades and a moving belt to control the thickness of the casted suspension (Figure 3A). Equipment for good dispersibility of the components and control of the film thickness are still the main limitations for scaling-up [19]. The method is useful when a very small amount of reinforcement is required, although being time and energy-consuming [106].

#### 3.4.2. Melt Processing

The melt processing has been a common option for the production of nanocellulosic composites, since it provides good production capacity, both in batch and continuous processing [61,62,63,70,106]. Basically, the polymer is melted and mixed with the NC in an extruder, for example a twin-screw extruder. The composite exits as extruded pellets or moulded by injection into an article (Figure 3B). In other words, the process involves direct mixing of the NC with molten polymer, optimizing the interaction between the two phases and avoiding the addition of solvents [106]. The nanocomposite is formed when the polymer—filler mixture is hardened above the glass transition temperature (Tg) of the polymer. The configuration of the extruder screws and the operating conditions (pressure and temperature in the different extruder zones) are crucial for obtaining a good distribution and dispersion of the filler within the matrix. Inadequate configuration may lead to the formation of cellulose aggregates in the matrix, as reported in the production of NFC-TPS composite (NFC gels with high water contents and starch powder) [70]. To facilitate the blending of hydrophilic (as nanocellulose) and hydrophobic components that hinders a good dispersion, the use of a master batch comprising NC and a polymer (PLA, PHA or PVA) is a common approach. This pre-mixture between the NC and a polymer (used as a carrier) have given good results regarding the degree of dispersion of NC within the matrix phase [62,106,108,109]. There is great potential in using melt processing due to its simplicity and direct mixing, without the use of solvents or water, and scale up capability [110]. However, this method has some disadvantages such as high energy consumption due to melting at high temperatures, required pressure and mixing. Also, most of the extruders for melt processing cannot be used with high water contents, which limits the potential of NC, due to hornification [14,111]—the nanofibers become tightly packed, due internal hydrogen bonds between adjacent surfaces, a process that occurs upon NC drying. Rewetting the cellulose has a very limited capability to reopen this arrangement [111]. Hence, the hornification of cellulose in large processing units causes a reduction on the accessible surface of NC and originates aggregates in the final composite. Additionally, during melt mixing, the shearing forces to obtain better dispersions between NCs and the matrix, may generate heat that causes the actual temperature of the melt to be higher than the set processing temperature [112]. Thus, all melting configuration process need to be carefully managed to prevent further degradation (controlled actual temperature and time of melt mixing). The incorporation of plasticized agents (such acetyl tributyl citrate or polyethylene glycol) is an effective way to lower the melting point of the composite, as also may improve stretchability of the final material [62,63].

#### 3.4.3. In Situ and Impregnation Methods

In this section two approaches used with BNC are addressed. More specifically, in situ (during fermentation) and ex situ (post-fermentation impregnation) process (Figure 3C). In both processes a 3D nanofibrillar BNC network naturally produced by static fermentation is used [16]. In the former method, the polymer is added to the culture medium as an additive to interact with BNC during its biosynthesis [16]. In the latter method, the polymer is added after BNC fermentation and washing. Never dried BNC membranes are immersed in a polymeric solution, to initiate the migration of the polymer into the bulk of the nanofibrillar network [16]. TPS and PHB are examples of polymers used to form composites with BNC by these methods [35,67,80].

The in situ process has some drawbacks: (i) the potential presence of culture medium residues; (ii) BNC-polymer interaction may be compromised due to the washing process; (iii) limitation of using antibacterial agents and the decrease of the BNC crystallinity and mechanical properties [113]. BNC-PHB composites, produced by an in situ process showed higher tensile strength (up to 217%) and higher Young modulus (up to 29%), than neat PHB [35]. Using an in situ process, BNC-TPS composites were produced using either corn or potato starch. The resulting films showed an increase in both tensile strength (up to 1144%) and Young modulus (up to 97%) [80].

Regarding the impregnation method, it should be noted that the “empty” spaces of wet BNC fibrillar network are at micrometric scale, therefore, only nano-sized compounds and polymers may migrate easily (but slowly) into the fibrillar network [113], such as emulsions systems comprising hydrophobic nanoparticles [113]. Despite these drawbacks, this method has found interest, namely in the development of functionalized BNC for intelligent and active packaging, to produce films [101,102] and freshness indicators [103,113,114] (see Section 3.3). Enhanced mechanical performance was reported for BNC-TPS composites in comparison to neat TPS films, with higher tensile strength (increased 137.1%) and higher Young modulus (increased 132%) [67].

The scalability of the aforementioned methods is however still very limited due to technical and economic factors. The limited size and (lack of) homogeneity of the BNC membrane and the processing in batch mode only, are constrains to increase process capacity [115]. Moreover, upstream, the high cost of the fermentation process and low BNC yields [49] are also important limitations.

#### 3.4.4. Layer-by-Layer Assembly (LbL)

The method of layer-by-layer (LbL) assembly mainly consists on the creation of multilayer films on solid supports as consecutive layer application in response to (commonly) electrostatic attraction of the materials in a solution or dispersion [116,117]. The layer deposition can occur by solution-dipping (as detailed in Figure 3D), spin or spray coating. The technique has the advantage of being simple, versatile, ensures good thickness control at nano scale level and the potential of coating 3D objects such as boxes, trays or cups [19,118], and allows the use of materials with different degrees of hydrophilicity, since the compatibility between hydrophilic and hydrophobic materials is attenuated [119,120]. Development of dense, ultra-thin nanocellulose layers in combination with different substrates, such as chitosan, nanoclays, PLA, PET, polyethyleneimine (PEI) and PVA, are described in the literature [18,19,117,118,119,120,121]. However, it is still far from being used industrially, since it is a slow process and is only suitable for small samples [122].

#### 3.4.5. Coating

The coating process can be defined as the application of a material onto a surface (Figure 3E), in which the solids form a film with good adhesion to the surface [14]. Coating is very common in food packaging and is used for all types of supporting materials, including cellulosic based materials. Most of the paper and board products used for food packaging contains a coating to improve moisture and grease resistance. There are several coating techniques used, such as solvent based coating, extrusion coating, aqueous dispersion, wax, hot melt coating and vacuum coating [122]. Some of the methods are reported to coat a NC layer, others to apply one or more layers where NC is a component of the coating.

Regarding nanocellulose-based coatings, solvent-based, extrusion and aqueous dispersion coating are the common techniques used. The excellent dispersibility of nanocellulose in water makes it very attractive as an aqueous coating that can be applied as a pure nanocellulose thin layer or as a composite with other traditional coating materials [19]. The use of nanocellulose as coating can inherently provide enhanced gas barrier and grease resistance to the substrates used [51,52,53,54,56,57,58].

A more recent coating technique is the application of nanofibers produced by electrospinning (Figure 3F) in the substrate. This methodology is based on high-voltage application in a polymer solution resulting on the formation of nanofibers through the electrostatic repulsion charges during spinning [123,124]. This technique is simple and cost effective, easy to set up on the process and continuous production (long fibres) [124,125]. In addition, the large surface area of the produced nanofibers allows to develop layers with high pore volume, different fibre length and different fibre diameter, with tunable mass transport properties [125]. However, solvent recovery problems, low productivity and instability are the disadvantages of an electrospinning set up [124]. Different electrospinning set ups for food packaging applications have been reported and a wide variety of polymers have been used: PLA, PVA, chitosan, polycaprolactone (PCL), poly(propylene carbonate (PPC), natural cellulose and starch [125]. However, few data are reported regarding the use of NC by electrospinning for food packaging applications. The electrospinning of BNC-PHB mixture was an efficient strategy to obtain high dispersion of BNC in hydrophobic matrices of polyhydroxy butyrate-co-hydroxy valerate (PHBV). The developed nanocomposite showed enhanced oxygen permeability (decreased 40% in comparison to neat PHBV) [50]. Also, the use of electrospun PVA-CNC nanofibres, improved dispersion of CNC within PLA matrix, which enhanced crystallinity degree of PLA and decreased oxygen permeability by 33% at high RH (50% and 75%) [126]. The electrospinning of PHB into both sides of nanopaper (composed of NFC and lignocellulose fibrils), produced a multilayer composite with high water resistance and low oxygen permeability [74]. Electrospinning is regarded as promising for combining nanomaterials with low compatibility, as nanocellulose and PHAs or PLA, without the need of NC modification.

#### 3.4.6. All-Cellulose Composites

All-cellulose composites (ACC) can be considered bio-derived monocomponent composites and refer to materials where the cellulosic phase is used both as reinforcing agents and as main matrix [127]. The main goal of developing all-cellulose composites is to improve the chemical bonding at the reinforcement–matrix interface. There are two approaches for the development of self-reinforced composites:

(i) Impregnation of previously fully dissolved cellulose into an undissolved cellulose matrix;
(ii) Selective dissolution where a cellulose matrix is partially dissolved and subsequently regenerated in situ, to create a matrix around the non-dissolved portion (Figure 3G);

Both strategies involve the dissolution of cellulose. The most common solvents used for dissolution are LiCL/DMAc, N-methylmorpholine N-oxide (NMMO), NaOH, acetone and ionic liquids (ILs) [128].

In the first approach, the dissolved NC is incorporated into the cellulose matrix, the solvent is removed, and cellulose is consequently regenerated and dried to form the ACC. In the work of Gindl et al. [129], BNC sheets were soaked in a cellulose acetate solution (with acetone as solvent). In the work of Puyol et al. [128] dissolved microcrystalline cellulose (MCC) (with trifluoroacetic acid and trifluoroacetic anhydride) was blended with dispersed NFC in chloroform. Both developed ACCs showed enhanced mechanical properties (tensile strength and Young modulus increased up to 400% and 173%, respectively with BNC) and transparency (higher than 80%) with the incorporation of nanocellulose [128,129].

Selective dissolution methods were also adopted to develop nanocellulose based ACCs. Nanofibres obtained from canola straw [130] and BNC [131] were partially dissolved with DMAc/LiCL at different dissolution times. In both studies, the produced ACC with partial dissolution time of 10 min, showed higher mechanical performance (ACC nanofibre canola straw [130]: tensile strength of 164 MPa and Young modulus of 15.2 GPa; BNC [131]: tensile strength of 411 MPa and Young modulus of 18 GPa).

The major drawback of the ACC approach lies on the use of solvents, with inherent cost and potential environment impact, because many solvents cannot be recycled (only NMMO has an industrialized recycling process) [132]. Additionally, and like the in situ and impregnation methods for BNC, this process may have some scale-up limitations (batch processing).

## 4. Safety of Nanocellulose Based Composites

In the current European legislation, all materials in contact with food (FCMs) must meet the requirements of the framework Regulation (EC) No 1935/2004. The regulation states that “Materials (…), shall be manufactured in compliance with good manufacturing practice and so that, (…) they do not transfer their constituents to food (…) in quantities which could: endanger human health; or bring about an unacceptable change in the composition of the food; or bring about a deterioration in the organoleptic characteristics thereof”. These requirements also apply to nanocellulose based composites, although this legislation does not address specific provisions for nanomaterials used in food contact materials. The regulation applicable to plastic materials—Regulation (EU) No. 10/201 as well as the Regulation (EC) No. 450/2009 on active and intelligent packaging materials indicate that a specific evaluation is required for substances in nanoform. Nanoparticles mechanisms of mass transfer and interaction with the host materials and with the food may be different from those known at the conventional particle size scale. Therefore, nanoparticles may lead to different exposure and different toxicological properties. Consequently, the pre-market authorisations which are based on the risk assessment of a substance with conventional particle size do not cover the use of the same substance in its nano-dimension which shall only be used if explicitly authorised and mentioned in the positive lists of the above-mentioned regulations [133].

The information relevant for the risk assessment of FCMs containing nanoparticles include three relevant aspects: (i) the characteristics of the nanomaterial used to produce the material; (ii) the characteristics of the material once it is incorporated into the FCM, as these may differ from the original characteristics, being influenced by the FCM matrix and/or the manufacturing conditions; and, (iii) the characteristics of any nanomaterial that migrates into the food matrix which is influenced by the food environment [134].

The nanoparticles size, shape and aggregation properties, among other factors, can affect the interactions of NC with living cells [135]. The safety of NC in food packaging depends on its transfer into food, which in turn determines the human exposure and on its toxicological profile. Different toxicity testing approaches are recommended to be applicable to nanomaterials depending on their migration behaviour and persistence in the nanoform, namely depending on if it occurs, or not, nanoparticle transformation into the non-nanoform in the food matrix before ingestion, or in the gastrointestinal tract following ingestion [134].

Cellulose and several derivatives are recognized as safe and are already authorised under the European Regulation (EC) No 10/2011 for plastics for food packaging, for use as polymer additive, production aid and other starting substances. Additionally, for food packaging, cellulose and cellulose acetate butyrate, different alkyl and hydroxyalkyl celluloses are authorised as additives and polymer production aids; and nitrocellulose and lignocellulose are authorised as monomers or other starting substances (Reg 10/2011). However, NC is not specifically listed and therefore it is not currently authorised for food contact applications.

There are few recent studies addressing the toxicity of NC. The endpoints recognised as most important regarding a nanomaterial toxicity are cytotoxicity, (pro-) inflammatory response, potential to generate reactive oxygen species (ROS) and oxidative stress, genotoxicity and integrity of the gastrointestinal barrier [134]. Endes et al. [136] reviewed key studies in vitro, in vivo and with ecosystem models regarding the biological impact of NC, addressing some of these endpoints. The compiled information revealed some inconsistency in the results achieved, as some studies showed low or no toxicity, while others stress the potential of NC for adverse effects. This was attributed to the variability of the biological systems, test conditions and source of the cellulosic material used, as well as to the lack of thorough characterization of the administered CNCs [136]. The physical-chemical characteristics, especially the aggregation level was considered to have a major impact on the results for inflammation. Some of the studies considered in this review, focused the inhalation route [137,138,139,140,141] which is of upmost relevance for occupational exposures as nano-sized particles (such NCs) may be inhaled during composite processing, but not for the use of NC-based composite food packaging. Therefore, studies with ingestion as the main route, focusing in relevant existing data gaps, such as migration and resulting exposure, uptake and fate in the gastrointestinal system and organ distribution, are still missing.

The effect of NC in the gastrointestinal tract was addressed in very few studies. A recent study evaluated the in vitro biological effect of unmodified and modified NC (carboxymethylation, phosphorylation, sulfoethylation and substitution with hydroxypropyltrimethylammonium) on human gut bacteria and gastrointestinal cells [142]. The metabolic activity and cell membrane integrity of intestinal cells Caco-2 and the growth of representative of microbiota bacteria were measured. Results indicated no cytotoxicity after exposure to unmodified NFC and to surface functionalized NFC. Furthermore, a bacteriostatic effect on *E. coli* was observed but not on *L. reuteri* [142].

Low in vitro (cell viability higher than 70%) cytotoxic effects and no mutagenic effect of TEMPO-oxidised NFC were reported in a recent study [143]. The cytotoxicity of the TEMPO-oxidised NFC was evaluated through the MTT test. However, the mutagenic activity was evaluated through the Ames test which is not considered suitable for nanomaterials owing to the inability of bacterial cells to internalise particles. Instead, mammalian cells are recommended to address genotoxicity endpoints [134,143]. The toxicological effects of ingested NC in in vitro intestinal epithelium and in vivo rat models were recently reported [144]. NFC and CNC, at 0.75% and 1.5% *w*/*w* were tested for the effect on cell layer integrity, cytotoxicity and oxidative stress. Micron-scale cellulose and TiO_2_ were used as controls. A 10% increase in ROS for 1.5% *w*/*w* CNC was reported, but no other significant changes in cytotoxicity, ROS or monolayer integrity were observed. Results from in vivo toxicity suggest that ingested nanocellulose has little acute toxicity and is likely non-hazardous when ingested in small quantities [144]. The authors highlight the need for chronic studies to assess long term effects, and potential detrimental effects of NC on the gut microbiome and absorbance of essential micronutrients. These studies would be of major interest for the application of nanocellulose in food packaging systems, where the chronic exposure is expected once the widely commercial use of NC will occur.

As with the synthetic counterparts, the migration to foods of intentionally added substances from the adjacent polymers and contaminants potentially present on the nanocellulose-based composite, must also be addressed. Besides contaminants and impurities, substances formed during nanocomposite processing, can appear throughout the production chain, when fossil-based and/or biopolymers are used. The processing of these materials may provide a source of non-intentionally added substances, with potential to migrate to foods upon contact. Potential contaminants from bio-based polymers include: heavy metals, persistent organic chemical contaminants, residues (e.g., pesticides, veterinary medicines), allergens and natural toxins [20]. Chemicals used in the pre-treatment of vegetable NC, by-products and culture media residues of BNC are also possible sources for chemical contaminants.

Studies reporting the impact of nanocellulose in composites on limiting the migration of components from the polymeric phase have been reported. The effect of incorporating CNC in several plastic films in the overall migration has been studied, particularly for biopolymers, such as PHB, PHBV and PLA. CNC-PHB films with different CNC loadings were tested for overall migration into ethanol 10% (*v/v*) and isooctane, respectively at 40 °C for 10 days and at 20 °C for 2 days. At low concentrations (1–2%) of CNC dispersed in the PHB matrix, the overall migration decreased in comparison to neat PHB, for both simulants. However, CNC loadings above 3% yield higher overall migration values, possibly due to a decrease in the adhesion between the hydrophilic CNC and hydrophobic PHB phases. The migration increased when the PHB was loaded with 5% CNC, from 20 to 40 µg/kg into isooctane and from 90 to 180 µg/kg into ethanol. The contact with ethanol simulant was reported to cause a change in the film appearance, in the thermal properties and also a decrease in the molecular weight due to the degradation of PHB chains into small oligomeric fractions [79]. The migration levels from a PHBV film into isooctane and 10% ethanol were also reduced by ca 50%, after incorporation of CNC into the plastic matrix [145].

The incorporation of NFC in PLA (ratio NFC:PLA 1:19) decreased by 20% the overall migration levels into ethanol 10%. However, no significant differences were found on the migration into the nonpolar simulant isooctane [146,147]. The overall migration values reported were low, in the order of 10 to 100 µg/kg. The results reported show that the chemical modification of the nanocellulose and the amount incorporated largely influence the migration behaviour. Both factors have an impact on the adhesion between the nanocellulose and the plastic, which may affect the simulant penetration and consequently the migration.

The CNC modification through esterification improved the compatibility with PLA, which reduced the migration from the PLA phase into both simulants [146]. This effect was observed for low CNC loading. However, increasing the CNC concentration (either modified and unmodified) in the composite led to an increase in the overall migration, particularly into isooctane [146]. As indicated, these studies focused on overall migration, and as such, characterisation of the migrants was not addressed. Studies focusing on the migration of specific substances have not been reported, apart from a few exceptions, and the influence of nanocellulose on the migration of substances of different chemical nature is not known. The migration of several hydrocarbons, as surrogate compounds for mineral oil, into the dry-food simulant Tenax^®^ was studied. A decrease of more than 90% was achieved by coating a HDPE film of 48 µm with a TEMPO-NFC 6 µm layer [148].

Clearly, there is a need for further research concerning the migration from nanocellulosic composites, particularly on screening throughout the production chain of nanocellulose based composites and traceability of possible contaminants throughout the production chain.

## 5. Technology Readiness Level of Nanocellulosic Composites

Parallel to the academic studies, an increasing growth has been noticed on the industrial production of NC and its composites. In fact, since 2010, 7636 patents are related to NC, 2827 patents related to nanocellulosic composites, 169 patents related to NC for food packaging applications and 29 patents related to NC-based composites for food packaging applications. This means that the interest in NC has been growing in the last decade, although not focusing particularly on the production of NC-based composites for food packaging applications. Additionally, most of the publications on NC-based composites for food packaging patents are from research institutions and universities. Nevertheless, several companies are currently commercializing vegetable NC (in the form of NFC and CNC) for a wide variety of applications (Table 2).

**Figure 4:**
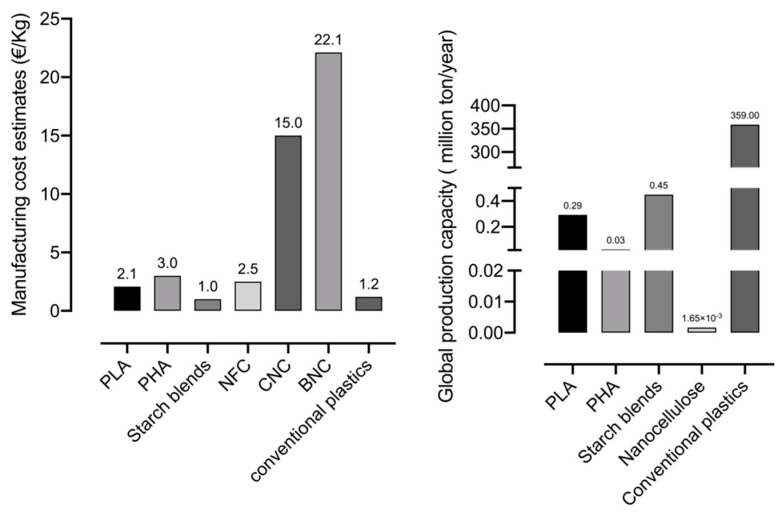
Global production capacity of bio-based and biodegradable materials; manufacturing cost estimates of bio-based materials, nanocelluloses (NFC, CNC and BNC) and conventional plastics (values adapted from [172,173,174,175,176,177,178,179,180]).

The high interest in NC, the advances in technology and scale up production can explain the advanced level of development. However, the price of NC is still volatile due to the production cost (related to the production method adopted), the market place and the specific format and characteristics [172]. The estimated production costs of CNC, NFC and BNC (Figure 4) are, respectively (in dry equivalent); 15, 2.15 and 22.11€/Kg, although the current market prices are higher [172,173,174,175]. It must be remarked that the cost of NCs in Figure 4 does not include hydrophobization, which may significantly add to the production cost taking in account the large surface area. On the other hand, this increase in cost may be somewhat mitigated by an increase in the production scale. Despite the good features of NC, the manufacturing cost (as that of PLA, PHA and starch) is still not competitive with petroleum-based plastics [156,176]. Nevertheless, there is margin to improve the market price of NC by increasing the production capacity (dry basis) (surrounding 1.6 thousand tonnes per year). The global production of NC was roughly estimated from the data collected from [177]. Achieving higher investments to improve technology processing and optimise the processes of NC production (especially for biotech BNC, still not available in large scale) is on demand. NFC has the lowest production cost since there is a higher investment by companies.

The TRL of thermoplastic biopolymers reinforced with natural fibres is at level 5 [156] demonstrating that there is an interest in technology development and validation. For the most promising bio-based materials, there is a need to lower the production cost and promote higher yields, through scalable processes for these materials become competitive in the market. Among the above-mentioned biopolymers (PLA, PHA and starch), combinations of starch and NFC are most likely to succeed, provided that the mixture is properly hydrophobized (e.g., treated with alkenylsuccinic anhydride). This combination turns out to be simple to process, as both are hydrophilic; and the alkenylsuccinic anhydride treatment will hydrophobize the composite to ensure enhanced water vapour permeability and water uptake, a known problem within NCs-TPS composites [82,181]. Another successful strategy to produce composites with two materials with low compatibility (as NCs and PLA) is the preparation of a master batch. This pre-mixing will assure a better dispersion within the matrix during melt mixing. A highly homogeneous composite is obtained with interesting mechanical and barrier properties [106,108,109].

Regarding specific applications to food contact materials and packaging there are already in the market some commercial solutions with biopolymer thermoplastics reinforced with natural fibre (Table 3) [182,183,184,185,186,187,188,189,190,191,192], although other applications, such as construction and automotive are more common. A few examples include FuturaMat which develops composites with BioFibra^®^ used to compostable coffee pods [185] and Kareline Oy that develops tableware based in Kareline^®^ [182]. In both cases the composites are based in PLA and wood fibre but do not incorporate NC in their product composition. Specific cases using NC in fibre based products are: (i) CelluComp (UK), which extract cellulose from root vegetables and produces Curran^®^ cellulose nano-fibres that can be used in coatings and paper and board for packaging [190], (ii) Stora Enso (Finland), which develops biocomposite and paperboard grades containing microfibrilated cellulose [191], claimed to be used in Elopak beverage cartons for milk [192]. Currently, the conventional cellulose based materials have a stronger presence (with NatureFlex and Cellophane from Futamura and PaperWise with Bio4pack) in the market (Table 3). So, NC related applications in general are on different TRL levels, and specifically regarding food packaging applications the TRL seems to be lower, calling for additional research investment.

## 6. Final Considerations

The purpose of this review was to assess the potential of using nanocellulose in the food packaging industry. Great amount of research was reported regarding the development of composites with different types of nanocellulose (NFC, CNC and BNC). Regardless of the matrix phase, a positive effect of nanocellulose was generally observed, namely improved the mechanical and oxygen properties, particularly at low humidity. On the other hand, low water resistance (vapour and liquid) is observed, which is an important feature for food packaging applications. Several approaches were reported to modify nanocellulose in order to counteract the poor water resistance, to promote better dispersion in hydrophobic matrices and to provide functionality, specifically for the application on active and intelligent packaging. The technologies of solvent casting, melt processing, impregnation, coating, layer by layer deposition, electrospinning and all-cellulose composites may be adopted for the production of NC composites, allowing the application of nanocellulose on several fronts of food packaging, as a reinforcing agent, as a barrier agent and for smart packaging.

Studies regarding the toxicity of nanocellulose and the impact of nanocellulose on the overall migration of composites, should be considered before its application in food contact materials, the available results indicating lack of or low toxicity. However, further research should be addressed to demonstrate the safety of nanocellulose, since it is not currently authorised for food contact applications. NC is produced industrially and commercially available, but its main applications lie on the composites and automotive industries.

The use of NC-based materials in replacement of petroleum-based plastics is still a major challenge, due to existing technological weaknesses regarding rheological behaviour, scalability and overall properties of the final material in regard to the requirements of today’s packaging systems. The sustainability of the global process of handling the biomatter and converting into packaging, such as the energy input and the use of solvents for example, should be considered and balanced with the impact of using reusable and recyclable, although non-renewable materials, together with safety and quality food requirements. Nevertheless, the potential for specific applications of functionalised nanocellulose is well recognised for specific and tailor-made high-valuable applications and these is expected to drive the research efforts in near future. Further research and investment on nanocellulose-based materials should be made in order to fill the narrowing regulatory, economic and technologic gap between the sustainable and the conventional packaging used in the food industry.

## Figures and Tables

**Table 1:** Barrier properties requirements for specific food products [25] and typical materials used.

Product	WVTR (g/m^2^.day) 23 °C	Oxygen Permeance (cm^3^_STP_/m^2^.day.Pa) 23 °C	Shelf-Life (Months)	Materials Typically Used
**Low moisture foods, a_w_ < 0.6**				
**Nuts, snacks, chips**	0.093–3.0	1.6 × 10^−6^–9.6 × 10^−5^	3–12	Metallised films, Laminates with EVOH, PP
**Coffee**	0.61–1.1	8.7 × 10^−6^–1.3 × 10^−5^	12–18	PP or PET metallised or AL foil laminates
**Other dried foods**	0.14–1.7	6.8 × 10^−7^–8.2 × 10^−6^	12–24	PP or PET metallised, Laminates with EVOH
**Oils**	<30	2.6 × 10^−5^–2.6 × 10^−4^	>12	PET, Glass
**High moisture foods, a_w_ > 0.9**				
**Beer**	1.4–3.0	4.5 × 10^−7^–2 × 10^−6^	6–12	Glass, PVDC- coated PET, Metal can
**Wine**	1.0–1.4	1 × 10^−6^–9.5 × 10^−6^	>12	Glass, PET, Bag in box
**Fruit juices, soft drinks**	0.47–12.2	6.1 × 10^−6^–6.14 × 10^−4^	1–18	Glass, PET, Metal cans, bag in box, Aseptic multilayer
**UHT milk**	2.7–5.3	3.5 × 10^−6^–5.6 × 10^−5^	2.5–5	Aseptic multilayer
**Hard cheese**	50	8.6 × 10^−4^–3.45 × 10^−3^	2	PP, HDPE
**Fats**	5.2–9.2	6.8 × 10^−5^–8.0 × 10^−4^	3	Fat resistant paper, PP
**Retorted food**	0.40–7.6	5.9 × 10^−6^–5.0 × 10^−5^	3–36	Metal can, Glass jar, Laminates: PET or PP with EVOH or polyamide
**Fresh foods**				
**Fruits, vegetables, fresh salads**	10–4000	1 × 10^−1^–2	0.25	LDPE, PP
**Meat and meat based products**	2–100	2 × 10^−4^–1 × 10^−1^	0.25–0.5	PS and PET trays
**Dairy products**	0.2–8	6 × 10^−4^–5 × 10^−2^	0.5	HDPE, PP, PS

**Table 2:** Nanocellulose producers.

NC Type	Companies	Applications
**CMF**	Celova [149]; Sappi [150]; Exilva [151]; FiberLean tecnologies [152]; FiloCell (Kruger company) [153]	Paper; Packaging; coatings; Paints; Cosmetics; Food; Filtration; Environmental remediation; Art Conservation; Adhesives, Agricultural chemicals; HI&I cleaning; Engineered applications; Polymer composites; Cement; Cosmetics; Sealants;
**NFC**	Sappi [147]; American Process [154]; US Forest products Lab [155]; Paperlogic [156]; University of Maine [157]; Nippon Paper [158]; Oji Paper [159], Innventia [160]; Cellulose Lab [161]; FiloCell (Kruger company) [153]	High-tech spun fibres; Antimicrobial films; Water absorbent pads in medical applications; Electronic displays; Food packaging; Flavour carrier; Suspension stabiliser; Thickener in food; Polymer composites; Cement; Paper; Cosmetics; Paints; Coatings; Sealants; Adhesives
**CNC**	American Process [154]; Melodea [162]; Innotech Alberta [163]; US Forest products Lab [155]; Blue Goose Biorefineries [164]; Celluforce [165]; Cellulose Lab [161]	Packaging, Paints; Coatings; Oil and Gas; Adhesives; Paper and non-wovens; Cement; Plastics; Composites; Cosmetics; Health Care; Food and Beverages; Electronics;
**BNC**	Cellulose Lab [161]; Bowil Biotech [166]; JeNaCell GmbH [167]; HYLOMORPH [168]; Weyerhaeuser [169]; Xylos [170]; Biofill [171]	Cosmetics, Biomedical,

**Table 3:** Cellulose-based products in the market

	Company	Products	Applications	Reference
**Thermoplastics reinforced with natural fibres**	Kareline	Natural fibres and plastics (injection moulding)	Tableware, appliances	[182]
	Scion	Natural fibre reinforced plastics (mainly PP) (injection moulding)	Automotive sector	[183]
	Fasal	Natural fibre reinforcing maize, natural or synthetic resins (injection moulding)	Toys;	[184]
	FuturaMat	BioFibra- derived from renewable ressources (biopolymers, wood flour and additives of vegetable origin) PolyFibra- Made from vegetable fibres and partially biobased;	Toys; horticulture; Agriculture; Pieces of equipment; Furnitures; Construction;	[185]
	GreenGran BV	PLA and PHB reinforced with natural fibres	Tools	[186]
	FkuR	Bio-based TPE with wood fibres	Soft-touch handles, toys, tools or sports equipment	[187]
**Cellulose based products**	Futamura	Cellophane Natureflex	Food Packaging	[188]
	Bio4Pack	Paperwise	Food Packaging	[189]
**NC based products**	Cellucomp	Curran^®^	Paints, coatings, Food and other packaging	[190]
	Storaenso	Microfibrilated cellulose based materials	Paper, Food and other packaging, intelligent packaging	[191]
	Elopak	Naturally Pure-Pak^®^	Food packaging	[192]
